# Predictive value of the non-high-density lipoprotein cholesterol to high-density lipoprotein cholesterol ratio (NHHR) for all-cause and cardiovascular mortality with non-hyperhomocysteinemia: evidence from NHANES 1999 to 2006

**DOI:** 10.3389/fnut.2025.1586558

**Published:** 2025-05-20

**Authors:** Liang Deng, Haicheng Zhong, Sheng Zhou, Ziming Wang, Jingjing Sun, Chang Liu, Xinghui Li, Quankai Cheng, Jie Deng

**Affiliations:** ^1^College of Traditional Chinese Medicine and Health Services, Shanxi Datong University, Datong, China; ^2^Department of Respiratory and Critical Care Medicine, The Second Affiliated Hospital of Xi'an Jiaotong University, Xi'an, China; ^3^Department of Cardiology, The Second Affiliated Hospital of Xi'an Jiaotong University, Xi'an, China; ^4^School of Public Health, Shaanxi University of Chinese Medicine, Xi'an, China

**Keywords:** NHHR, NHANES, NHHcy, mortality, CVD

## Abstract

**Background:**

The non-high-density lipoprotein cholesterol to high-density lipoprotein cholesterol ratio (NHHR) has emerged as a promising biomarker for lipid metabolism, with established links to mortality in various chronic diseases. However, its prognostic value in individuals with non-hyperhomocysteinemia (NHHcy), a population often overlooked in cardiovascular risk assessment, remains unexplored. This study aimed to determine the association between NHHR and all-cause and cardiovascular disease (CVD) mortality and its predictive value among adults with NHHcy.

**Methods:**

This study used the National Health and Nutrition Examination Survey database (1999–2006) and employed the National Death Index to determine mortality outcomes. The relationship between NHHR and mortality was evaluated using restricted cubic splines (RCS) and a multivariable Cox proportional hazards model. The threshold effects were assessed using a piecewise Cox proportional hazards model. Analysis of subgroups, interactions, and survival was done. Lastly, the NHHR's predictive value for mortality as well as its potential mediating effects were evaluated.

**Results:**

A total of 13,847 participants were included in our study. Over the course of the follow-up, there were a total of 2,886 mortality, of which 739 were due to CVD. A “U-shaped” correlation between NHHR and mortality was shown by the RCS. A one-unit rise in NHHR was linked to a 19 and 17.4% decrease in the odds of all-cause and CVD mortality, respectively, when NHHR was below the inflection point; the hazards rose by 6.4 and 11.9%, respectively, when NHHR was over the inflection point. There were interaction effects between several subgroups in the relationship between NHHR and mortality from all-cause and CVD. Additionally, NHHR's area under the curve for predicting death from all-cause and CVD was 0.897 (0.890–0.904) and 0.921 (0.910–0.932), respectively. According to mediation analysis, the association between NHHR and all-cause mortality was mediated by aspartate aminotransferase.

**Conclusion:**

NHHR was shown to have a “U-shaped” relationship with both all-cause and CVD mortality in the NHHcy population, as well as an excellent mortality predictive value. In the future, NHHR could be used for predicting mortality risk and prognosis in the NHHcy population.

## 1 Introduction

Cardiovascular diseases (CVD) have grown to be a major burden on human health and the worldwide economy, with their incidence and mortality rates steadily rising, presenting a significant obstacle to public health worldwide (1). Recent studies indicate that CVD persists as the primary cause of age-standardized worldwide mortality, accounting for 235.18 deaths per 100,000 people (2). In light of this, identifying persons at high cardiovascular risk early on is essential to successfully lowering the incidence and death rates of these conditions.

Hyperhomocysteinemia (HHcy) is a metabolic disease linked to increased blood levels of homocysteine (Hcy), with a global prevalence of approximately 5–7% (3, 4). Numerous investigations have demonstrated a close correlation between HHcy and the development and course of a number of chronic illnesses, including dyslipidemia, atherosclerosis, and osteoporosis (5–7). The causes of HHcy include genetic factors, chronic diseases, and dietary factors. Treatment typically involves supplementation with vitamins B6, B12, and folate (8, 9). Research by Li et al. (10) and Liu et al. (11) has indicated that after implementing effective measures to control Hcy levels, the risk of CVD significantly decreased. Therefore, controlling Hcy levels holds significant clinical value (3).

The risk of all-cause and CVD mortality in populations with diabetes or hypertension has been evaluated using general systemic immune-inflammatory markers, such as the neutrophil-lymphocyte ratio (12–14). In recent years, the clinical value of non-high-density lipoprotein cholesterol (NHDL-C) has been widely recognized, and the National Institute for Health and Clinical Excellence had recommended replacing LDL cholesterol with NHDL-C as the main target for CVD risk reduction in diabetic patients in 2021 (15). Thus, the ratio of NHDL-C to high-density lipoprotein cholesterol (HDL-C) (NHHR) is gaining attention as an emerging composite lipid marker. Compared with traditional lipid markers, NHHR integrates the atherogenic lipid component (NHDL-C) and the protective lipid component (HDL-C) in a single ratio, which better reflects the balance between atherogenic and anti-atherogenic, especially in patients with metabolic syndrome and high-risk populations, and can help researchers better stratify cardiovascular risk (16–18). In addition, the calculation of NHHR requires only routine lipid profile data, which is easily accessible and has wide applicability in clinical work. It may be used to diagnose a number of conditions, such as diabetes, gallstones, and CVD (19–21). The study by Yu et al. (15) indicates that in adults with diabetes or pre-diabetes, NHHR was linked to mortality. Su et al. (22) also found that NHHR is a potential predictor of mortality in hypertensive patients. However, for populations with normal Hcy levels, their health status is often overlooked, and thorough studies investigating the predictive significance of NHHR in patients with non-hyperhomocysteinemia (NHHcy) have not yet been conducted.

We utilized information from the National Health and Nutrition Examination Survey (NHANES) 1999–2006 to close this gap. The primary goal was to determine the inherent correlation between NHHR and all-cause and CVD mortality in NHHcy population, and to further explore the correlation between the ratio and various population characteristics, with the intention of offering helpful advice for creating successful preventative measures.

## 2 Materials and Methods

### 2.1 Research data

**Figure 1:**
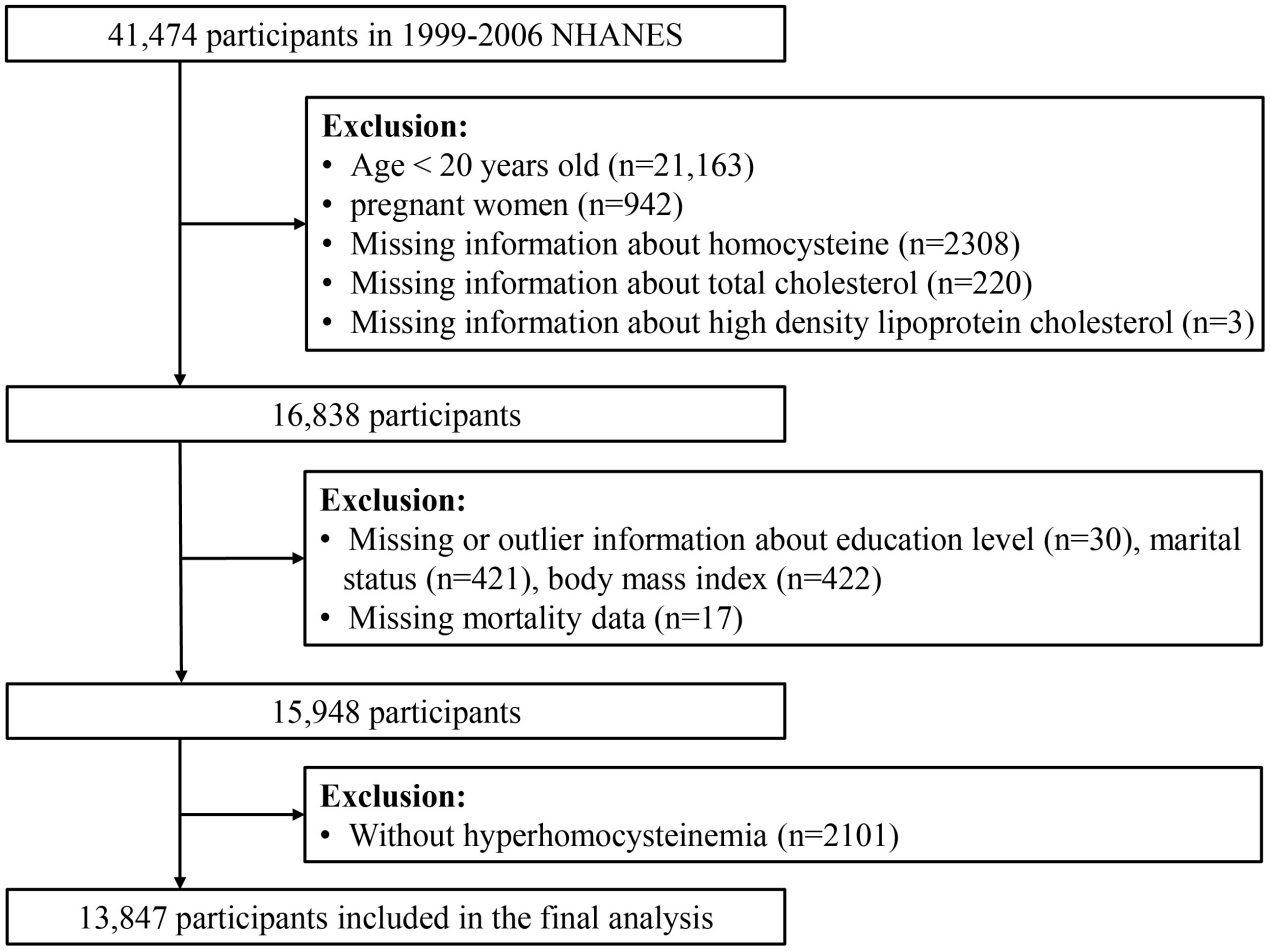
Flowchart of the participants' selection from NHANES 1999 to 2006.

An important epidemiological survey study, the NHANES was carried out by the Department of Health and Human Services' National Center for Health Statistics. All participants completed written informed consent forms, and the ethics committee approved the survey (23). Researchers that wish to do different health research and policy-making can ask for access to the database. To investigate the relationship among NHHcy individuals, we mainly used data from 1999 to 2006. Initially, 41,474 participants were included. We eliminated persons < 20 years old, 942 pregnant women, and 2,038 and 223 participants who were not included due to missing Hcy and cholesterol data, respectively. Additionally, 890 individuals with missing baseline demographic data and 2,101 individuals who did not have HHcy were excluded. Finally, the study comprised 13,847 individuals (Figure 1).

### 2.2 Definition of NHHR

As is well known, lipid parameters are a crucial component of laboratory evaluations for CVD. These parameters include various measurements, such as total cholesterol (TC), low-density lipoprotein cholesterol (LDL-C), HDL-C, and triglycerides (18). The ratio of NHDL-C to HDL-C, where NHDL-C is determined by deducting HDL-C from TC levels, is known as NHHR. It is important to note that the calculation of NHHR relies heavily on TC and HDL-C, which, according to the most recent international guidelines, remain relatively stable in both the fasting and non-fasting states (24). Therefore, the measurement of NHHR is minimally affected by the fasting state, making it a reliable indicator in clinical and epidemiologic studies. In the NHANES program, participants were asked to collect blood samples after an overnight fast to further minimize fluctuations induced by recent food intake, thus ensuring data rigor. The blood samples were then analyzed using a Roche Cobas 6000 and Modular P analyzer in accordance with international guidelines. All assays were obtained by standardized enzyme analysis under strict quality control protocols (25).

### 2.3 Definition and assessment of mortality

The mortality statistics utilized in this study were obtained from the National Death Index (NDI) website. Using probabilistic matching methods, this website combines survey respondent data with NDI death certificate information, which is updated through December 31, 2019 (15). All-cause mortality refers to the total sum of all-cause of mortality, while the international classification of diseases, 10th Revision (ICD-10) is used to classify CVD mortality; pertinent codes are I00-I09, I11, I13, I20-I25, and I26-I51 (26).

### 2.4 Additional covariates

We cited earlier research and took into account any confounding factors that could have an impact on the findings (15, 21). These mainly fall into four categories: demographic, examination, laboratory data, and lifestyle variables. Missing covariate data were imputed using the “jomo” package in R (27). After 1,000 iterations, 10 filled datasets are generated to ensure random independence between the datasets. This package can achieve multi-layer joint modeling under the assumption of random missing, ensuring good interpolation quality (28). Supplementary Table S1 lists the precise definitions of variables, and further information is available at https://wwwn.cdc.gov/nchs/nhanes/.

### 2.5 Statistical analysis

We separated the individuals into four groups according to the NHHR quartiles since the NHANES experiment used a weighted sample statistical technique. We used the weighted student's *t*-test to evaluate continuous data and the weighted chi-square test to evaluate categorical ones. Categorical data are reported as percentages, whereas continuous variables are shown as means with standard errors (SE) or medians with first and third quartiles. To examine the relationship between NHHR and mortality, we developed three different regression models. Regression analysis utilizing multivariable Cox proportional hazards was used to analyze the relationships, and the possible nonlinear link between NHHR and mortality was addressed using restricted cubic splines (RCS). By setting multiple nodes and allowing continuous variables to show smooth nonlinear trends, RCS is able to more accurately identify potential nonlinear correlations such as “U-type” and “J-type”, and avoid the bias caused by linear assumptions, thus significantly improving the model's goodness-of-fit and biological explanatory ability (29). In addition, multicollinearity tests were conducted for all variables included in regression analysis. In order to investigate the variations across different groups, we also performed subgroup analysis. For survival analysis, the Kaplan-Meier approach was also employed to evaluate the survival probability of various NHHcy patient groups according to NHHR levels. Finally, we performed a 10-fold cross-validation using least absolute shrinkage and selection operator (LASSO) regression, with the best features selected at λ.1se (21). This regularization technique automatically identifies the most predictive features from high-dimensional data while effectively addressing multicollinearity issues, thereby constructing a parsimonious risk prediction model with enhanced stability (30). Subsequently, NHHR was incorporated with the LASSO-selected features to develop the predictive model. The model's clinical worth was rigorously evaluated through receiver operating characteristic (ROC) curve analysis, decision curve analysis (DCA), and calibration curve. Additionally, 1,000 bootstrap sampling simulations were performed using the nonparametric percentile method using the R “mediation” software package, and the bias-corrected bootstrap technique was used to determine its confidence intervals to estimate the moderating effect of aspartate aminotransferase (AST), which balances computational efficiency while ensuring robust results (31). Data analysis was conducted using R software (version 4.3.3), and *P* < 0.05 was considered statistically significant (two-sided).

## 3 Results

### 3.1 Characteristics of participants

This research had 13,847 individuals in total. Table 1 lists the demographic and other covariate data of the participants, who were split up into NHHR quartiles. The participants were 47.95 years old on average, with 48.44% male and 51.56% female. The bulk of factors showed significant variations between the various NHHR quartiles, according to statistical analysis. Participants in higher NHHR quartiles often older, with greater body mass index (BMI) and blood pressure, less physical activity, and a higher likelihood of having CVD. Additionally, the majority of participants were male, and a higher proportion of them smoked and consumed alcohol.

**Table 1:** Baseline characteristics of subjects by quartiles of NHHR in NHANES 1999–2006.

**Characteristic**	**Quartiles of NHHR**				***P*-value**
	**Q1 (≤2.07)**	**Q2 (2.07–2.86)**	**Q3 (2.86–3.88)**	**Q4 (≥3.88)**	
Age (years)	46.00 ± 19.07	47.70 ± 18.48	49.09 ± 17.63	49.02 ± 16.20	**< 0.001**
AST (U/L)	24.51 ± 12.14	25.12 ± 32.24	25.52 ± 18.64	26.72 ± 20.16	**< 0.001**
TC (mmol/L)	4.59 ± 0.89	4.94 ± 0.90	5.29 ± 0.88	5.95 ± 1.13	**< 0.001**
HDL-C (mmol/L)	1.79 ± 0.41	1.43 ± 0.27	1.23 ± 0.21	1.00 ± 0.19	**< 0.001**
Folate serum (nmol/L)	33.30 ± 22.54	32.53 ± 20.63	32.34 ± 22.21	31.31 ± 32.68	**0.011**
Folate erythrocyte (nmol/L)	662.86 ± 298.83	665.00 ± 288.84	675.21 ± 306.44	664.55 ± 273.76	0.280
Vitamin B12 (IQR, pmol/L)	369.74 (278.96, 492.98)	356.45 (271.58, 480.44)	357.19 (276.75, 459.04)	348.34 (268.07, 455.35)	**< 0.001**
Mean SBP (mmHg)	121.99 ± 19.83	123.75 ± 19.38	125.38 ± 19.43	126.85 ± 18.28	**< 0.001**
Mean DBP (mmHg)	68.94 ± 12.44	69.88 ± 12.66	71.38 ± 13.23	73.57 ± 12.86	**< 0.001**
HCY (umol/L)	7.63 ± 1.91	7.78 ± 1.90	7.99 ± 1.90	8.09 ± 1.81	**< 0.001**
Follow-up period (months)	181.22 ± 49.30	183.92 ± 48.79	185.46 ± 48.79	186.49 ± 50.5	**< 0.001**
Gender, *n* (%)					**< 0.001**
Male	1,110 (32.06)	1,437 (41.57)	1,847 (53.29)	2,314 (66.84)	
Female	2,352 (67.94)	2,020 (58.43)	1,619 (46.71)	1,148 (33.16)	
Race/ethnicity, *n* (%)					**< 0.001**
Mexican American	611 (17.65)	746 (21.58)	895 (25.82)	936 (27.04)	
Other Hispanic	113 (3.26)	142 (4.11)	172 (4.96)	164 (4.74)	
Non-Hispanic White	1,740 (50.26)	1,719 (49.73)	1,627 (46.94)	1,772 (51.18)	
Non-Hispanic Black	867 (25.04)	729 (21.09)	652 (18.81)	460 (13.29)	
Other race	131 (3.78)	121 (3.50)	120 (3.46)	130 (3.76)	
Education level, *n* (%)					**< 0.001**
Less than high school	855 (24.70)	963 (27.86)	1,074 (30.99)	1,212 (35.01)	
High school or equivalent	771 (22.27)	842 (24.36)	837 (24.15)	844 (24.38)	
College graduate or above	1,836 (53.03)	1,652 (47.79)	1,555 (44.86)	1,406 (40.61)	
Marital status, *n* (%)					**< 0.001**
Married or living with a partner	1,968 (56.85)	2,096 (60.63)	2,287 (65.98)	2,382 (68.80)	
Widowed/divorced/separated	724 (20.91)	728 (21.06)	671 (19.36)	641 (18.52)	
Never married	770 (22.24)	633 (18.31)	508 (14.66)	439 (12.68)	
PIR, *n* (%)					**< 0.001**
< 1.3	780 (22.53)	869 (25.14)	894 (25.79)	959 (27.70)	
1.3–3.5	1,429 (41.28)	1,405 (40.64)	1,462 (42.18)	1,444 (41.71)	
≥3.5	1,253 (36.19)	1,183 (34.22)	1,110 (32.03)	1,059 (30.59)	
BMI, *n* (%)					**< 0.001**
< 25 kg/m^2^	1,850 (53.44)	1,254 (36.27)	803 (23.17)	502 (14.50)	
25–30 kg/m^2^	963 (27.82)	1,142 (33.03)	1,337 (38.57)	1,436 (41.48)	
≥30 kg/m^2^	649 (18.75)	1,061 (30.69)	1,326 (38.26)	1,524 (44.02)	
Smoking status, *n* (%)					**< 0.001**
Yes	1,479 (42.72)	1,589 (45.96)	1,637 (47.23)	1,883 (54.39)	
No	1,983 (57.28)	1,868 (54.04)	1,829 (52.77)	1,579 (45.61)	
Alcohol consumption, *n* (%)					**0.002**
Yes	2,454 (70.88)	2,336 (67.57)	2,390 (68.96)	2,470 (71.35)	
No	1,008 (29.12)	1,121 (32.43)	1,076 (31.04)	992 (28.65)	
Diabetes, *n* (%)					**< 0.001**
Yes	225 (6.50)	290 (8.39)	325 (9.38)	386 (11.15)	
No	3,205 (92.58)	3,127 (90.45)	3,088 (89.09)	3,009 (86.92)	
Prediabetes	32 (0.92)	40 (1.16)	53 (1.53)	67 (1.94)	
Hypertension, *n* (%)					**< 0.001**
Yes	1,123 (32.44)	1,268 (36.68)	1,377 (39.73)	1,442 (41.65)	
No	2,339 (67.56)	2,189 (63.32)	2,089 (60.27)	2,020 (58.35)	
Cardiovascular disease, *n* (%)					0.083
Yes	212 (6.12)	263 (7.61)	252 (7.27)	252 (7.28)	
No	3,250 (93.88)	3,194 (92.39)	3,214 (92.73)	3,210 (92.72)	
Physical activity, *n* (%)					**< 0.001**
Yes	1,887 (54.51)	1,733 (50.13)	1,616 (46.62)	1,518 (43.85)	
No	1,575 (45.49)	1,724 (49.87)	1,850 (53.38)	1,944 (56.15)	
Stroke, *n* (%)					**0.015**
Yes	74 (2.14)	72 (2.08)	106 (3.06)	99 (2.86)	
No	3,388 (97.86)	3,385 (97.92)	3,360 (96.94)	3,363 (97.14)	
Mortality, *n* (%)					**0.028**
Yes	672 (23.28)	718 (24.88)	722 (25.02)	774 (26.82)	
No	2,790 (25.45)	2,739 (24.99)	2,744 (25.03)	2,688 (24.52)	

Continuous variables: values are expressed as mean ± standard error or median (Q1, Q3).

Categorical variables: values are expressed as numbers (percentage).

AST, aspartate aminotransferase; TC, total cholesterol; HDL-C, high density lipoprotein cholesterol; SBP, systolic blood pressure; DBP, diastolic blood pressure; HCY, homocysteine; HHcy, hyperhomocysteinemie; PIR, poverty income ratio; BMI, body mass index. Bold P values represent statistical significance (*P* < 0.05, two-sided).

### 3.2 Relationship between NHHR and mortality

We used multivariable Cox analysis to look into the relationship between NHHR quartiles (from lowest to highest: Q1, Q2, Q3, and Q4) and mortality (Table 2). According to the results, as compared to Q1, each unit increase in NHHR level for people in Q3 was associated with a 15% reduction in all-cause mortality [HR 0.85 (0.76–0.94)] and a 22% reduction in CVD mortality [HR 0.78 (0.63–0.97)] in Model 2. The similar outcome was demonstrated in Model 3, where persons in Q3 saw a 17% drop in all-cause mortality [HR 0.83 (0.75–0.93)] and a 25% decrease in CVD mortality [HR 0.75 (0.60–0.93)] for each unit increase in NHHR level. Trend analysis further showed that this association was statistically significant (*p* for trend ≤ 0.05).

**Table 2:** HRs (95% CIs) for all-cause and cardiovascular mortality according to NHHR quartiles.

**Characteristic**		**Model 1** ^**a**^		**Model 2** ^**b**^		**Model 3** ^**c**^	
		**HR (95% CI)**	***P*** **-value**	**HR (95% CI)**	***P*** **-value**	**HR (95% CI)**	***P*** **-value**
**All-cause mortality**							
NHHR	Quartile 1	Ref		Ref		Ref	
	Quartile 2	1.05 (0.94, 1.16)	0.384	0.92 (0.83, 1.03)	0.148	0.91 (0.82, 1.01)	0.076
	Quartile 3	1.04 (0.93, 1.15)	0.486	0.85 (0.76, 0.94)	**0.002**	0.83 (0.75, 0.93)	**< 0.001**
	Quartile 4	1.10 (0.99, 1.22)	0.064	0.98 (0.88, 1.10)	0.783	0.93 (0.84, 1.04)	0.225
	P for trend	1.03 (1.00, 1.06)	0.079	1.00 (0.96, 1.03)	0.872	0.94 (0.91, 0.98)	**0.002**
**Cardiovascular mortality**							
NHHR	Quartile 1	Ref		Ref		Ref	
	Quartile 2	1.01 (0.82, 1.25)	0.889	0.92 (0.75, 1.14)	0.441	0.92 (0.74, 1.13)	0.425
	Quartile 3	0.98 (0.80, 1.21)	0.886	0.78 (0.63, 0.97)	**0.023**	0.75 (0.60, 0.93)	**0.009**
	Quartile 4	1.14 (0.93, 1.40)	0.200	1.07 (0.87, 1.32)	0.541	0.98 (0.79, 1.22)	0.872
	P for trend	1.04 (0.98, 1.11)	0.192	1.02 (0.95, 1.10)	0.524	0.92 (0.86, 0.98)	**0.033**

95% CI: 95% confidence interval.

^a^Model 1: no covariates were adjusted.

^b^Model 2: adjusted for gender, age, and race.

^c^Model 3: gender, age, race, education level, marital status, PIR, BMI, smoking status, alcohol consumption, diabetes, hypertension, cardiovascular disease, physical activity, stroke, AST, folate serum, folate erythrocyte, vitamin B12, HCY.

HR, Hazard ratio; PIR, poverty income ratio; BMI, body mass index; AST, aspartate aminotransferase; RBC, red blood cell; HCY, homocysteine. Bold P values represent statistical significance (*P* < 0.05, two-sided).

### 3.3 Assessment of multicollinearity of variables

We assessed multicollinearity between exposure variables as well as other covariates using variance inflation factor (VIF) analysis. The results showed that the VIF values of several variables, including NHHR, were below 5 (Supplementary Table S2). Specifically, a VIF equal to 1 indicates that there is no multicollinearity among the factors; between 1 and 5 indicates that there may be correlation but not enough to be of undue concern; between 5 and 10 indicates that there may be a high degree of correlation; and a value >10 can be considered to have too high a degree of multicollinearity and the model is relatively unstable (15). This suggests that there is no significant problem of multicollinearity in the model of this study.

### 3.4 Exploring nonlinear relationship

**Figure 2:**
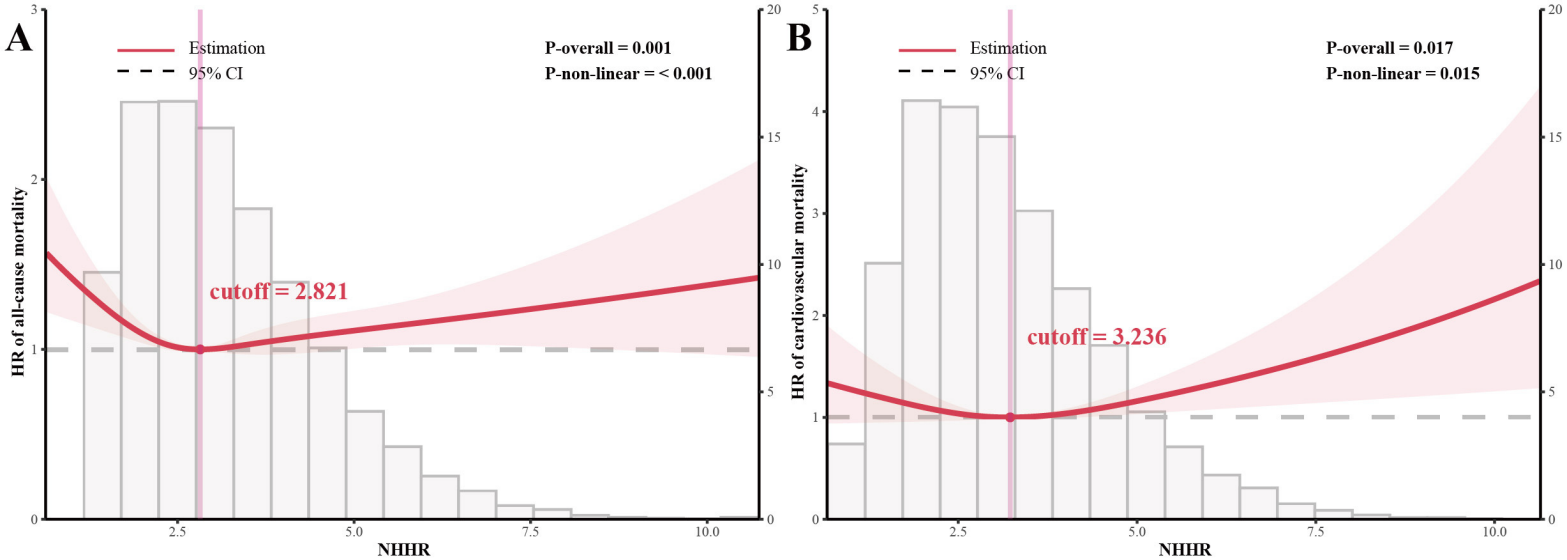
Restricted cubic spline bars were used to assess potential nonlinear associations between NHHR and risk of all-cause mortality **(A)** and CVD mortality **(B)** in the NHhcy population.

We built a Cox proportional hazards model with RCS to examine the relationship between mortality risk and NHHR in order to look for any nonlinear trends. NHHR was shown to have a substantial “U-shaped” nonlinear dose-response association with both all-cause (*p* < 0.001) and CVD (*p* = 0.015) mortality risk, according to the RCS regression model that was adjusted for all confounding variables (Figure 2). This prompted further investigation, and in the subsequent segmented Cox proportional hazards regression and threshold analysis (Table 3), we determined that the NHHR's critical threshold values for the risk of all-cause and CVD mortality were, respectively, 2.821 and 3.236. In particular, one unit increase in NHHR over 2.821 is associated with a 6.4% increase in risk of all-cause mortality [HR 1.064 (1.025–1.104)], whereas each unit rise below 2.821 is associated with a 19% decrease in risk [HR 0.810 (0.747–0.879)]. For CVD mortality, each unit increase in NHHR lowers the risk by 17.4% [HR 0.826 (0.729–0.936)] below the threshold, but each unit increase raises the risk by 11.9% [HR 1.119 (1.037–1.207)] over the threshold.

**Table 3:** Threshold effect analysis of NHHR on all-cause mortality and cardiovascular mortality in non-hyperhomocysteinemia participants.

**Adjusted models^a^**	**HR (95% CI)**	***P*-value**
**All-cause mortality**		
Model 1: Standard cox regression model	0.994 (0.965–1.023)	0.661
Model 2: Segmented cox proportional hazards model		
Inflection point	2.821	
NHHR < 2.821	0.810 (0.747–0.879)	**< 0.001**
NHHR ≥ 2.821	1.064 (1.025–1.104)	**0.001**
P for log-likelihood ratio test^b^		**< 0.001**
**Cardiovascular mortality**		
Model 1: Standard cox regression model	1.007 (0.951–1.065)	0.817
Model 2: Segmented cox proportional hazards model		
Inflection point	3.236	
NHHR < 3.236	0.826 (0.729–0.936)	**0.003**
NHHR ≥ 3.236	1.119 (1.037–1.207)	**0.004**
P for log-likelihood ratio test^b^		**< 0.001**

95% CI: 95% confidence interval.

^a^Adjusted models: gender, age, race, education level, marital status, PIR, BMI, smoking status, alcohol consumption, diabetes, hypertension, cardiovascular disease, physical activity, stroke, AST, folate serum, folate erythrocyte, vitamin B12, HCY.

^b^Model 2 compared to model 1.

HR, Hazard ratio; PIR, poverty income ratio; BMI, body mass index; AST, aspartate aminotransferase; HCY, homocysteine. Bold P values represent statistical significance (*P* < 0.05, two-sided).

### 3.5 Subgroup and interaction analysis

**Figure 3:**
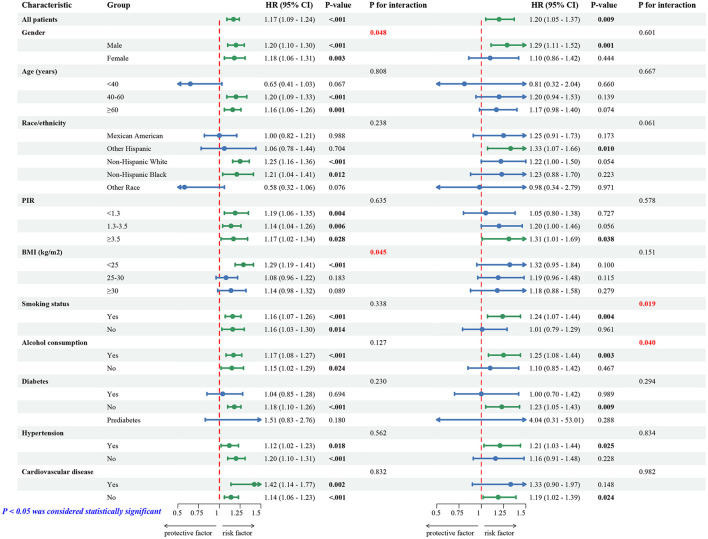
Subgroup and interaction analysis between NHHR and risk of all-cause and CVD mortality.

We used multivariable Cox proportional hazards regression and participant stratification by gender, age, race, and other factors to better understand the association between NHHR and all-cause and CVD mortality (Figure 3). The findings showed that different subgroups had different associations between NHHR and the risk of all-cause and CVD mortality (*p*-interaction < 0.05). In particular, the subgroups of gender and BMI showed significant interaction effects with all-cause mortality, whereas the subgroups of smoking status and alcohol consumption showed significant interaction effects with CVD mortality risk. Supplementary Figure S1 further visualizes these interaction effects.

### 3.6 Survival analysis

**Figure 4:**
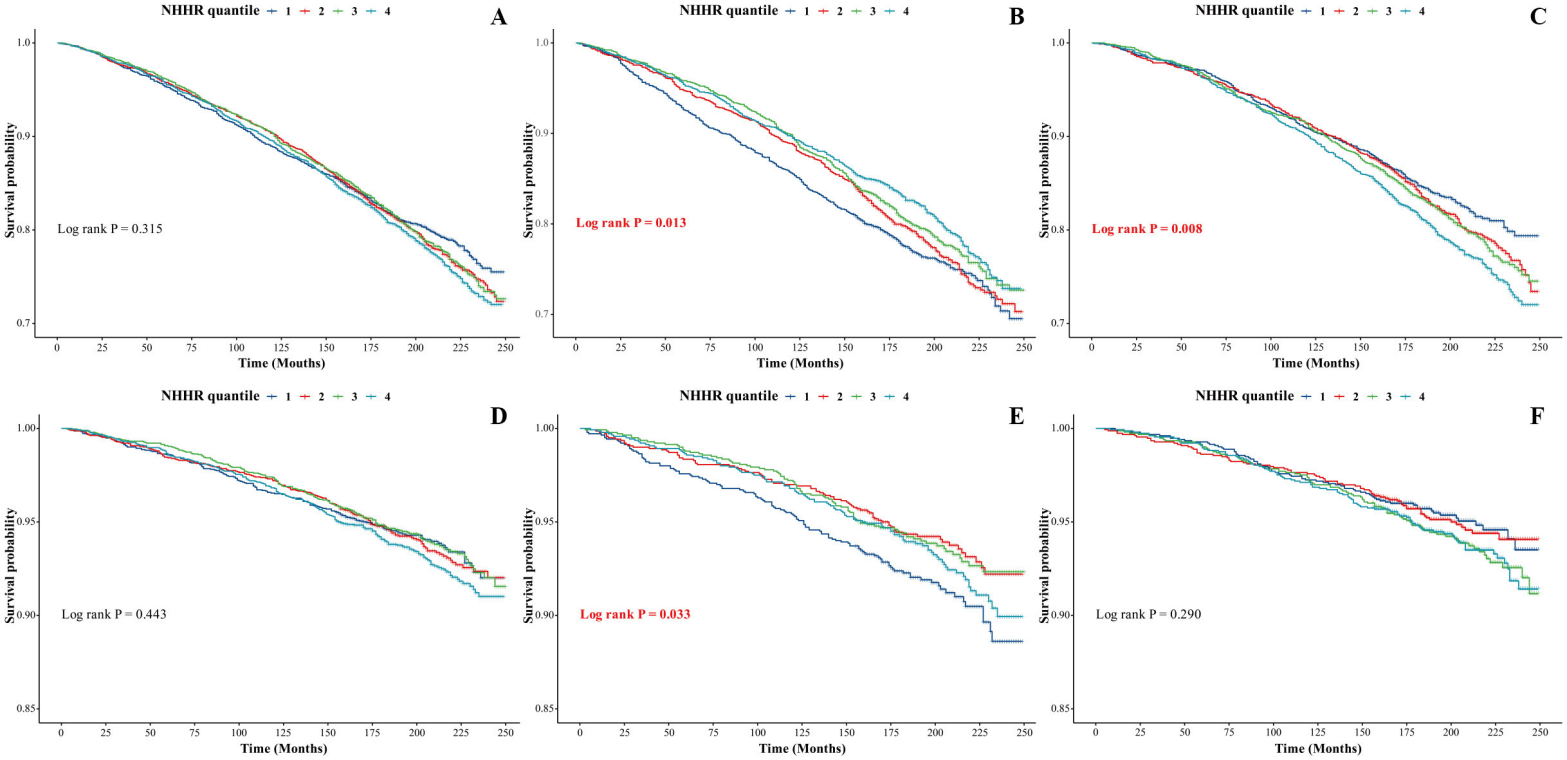
Multivariate Kaplan-Meier curves. **(A)** Association between NHHR and all-cause mortality risk in the whole population; **(B)** in the male group; and **(C)** in the female group. **(D)** Association between NHHR and risk of death from CVD mortality risk in the whole population; **(E)** in the male group; and **(F)** in the female group. The curves with different colors represent NHHR levels in different tertiles.

Over the course of the 250-month follow-up, 2,886 deaths were occurred, of which 739 were related to CVD. NHHR quartiles and all-cause or CVD mortality did not significantly correlate, according to the survival curves produced by the Kaplan-Meier technique (Figures 4A, D). Considering those differences in hormone, individual fat and muscle, and drug metabolism between male and female groups may affect lipid metabolism and thus the association of NHHR with adverse events. We further stratified participants by sex. As a result, we were surprised to find that, in the male group, significantly greater risks of all-cause and CVD mortality were linked to decreased NHHR values (*P* = 0.013, Figure 4B; *P* = 0.033, Figure 4E). In the female group, greater NHHR values were significantly associated with a greater risk of all-cause mortality, but no meaningful trend was observed in the risk of CVD mortality (*P* = 0.008, Figure 4C; *P* = 0.290, Figure 4F).

### 3.7 LASSO regression and ROC curves

**Figure 5:**
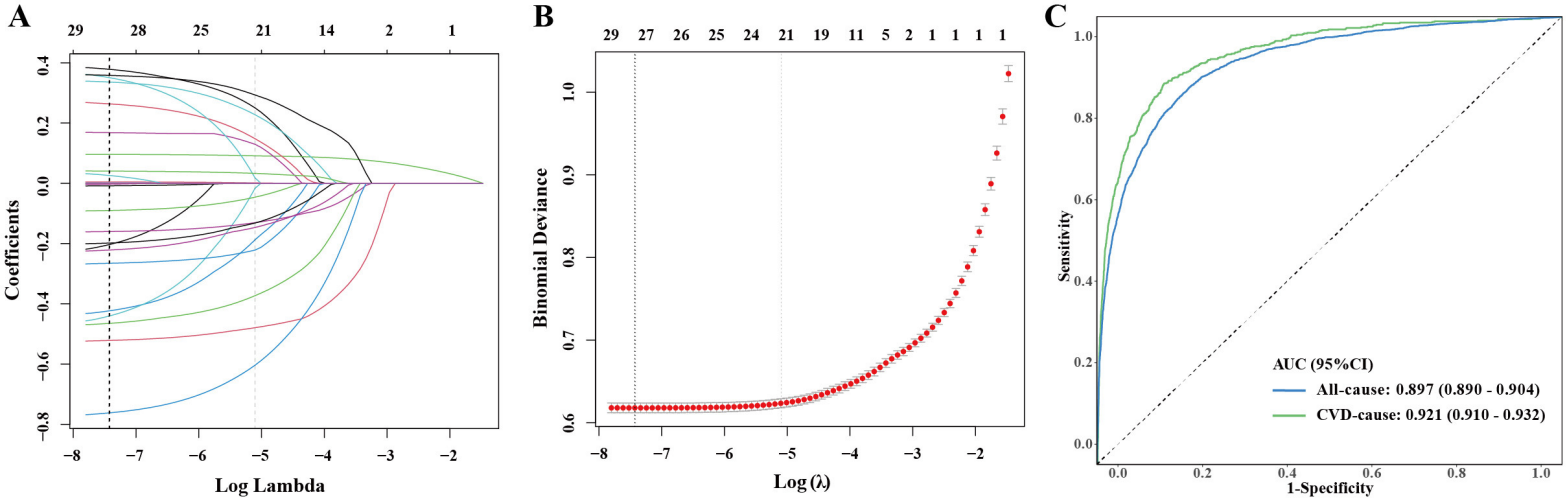
LASSO regression analysis was used to screen the factors most associated with the risk mortality and used as a basis for predictive modeling. **(A)** Plot of LASSO regression coefficients; **(B)** cross-validation plot; and **(C)** ROC curve used to assess the efficacy of the prediction model for diagnosing mortality risk in this study.

In order to build the forecasting model, LASSO regression was used for feature selection. In the end, 13 relevant variables were selected (log of best λ = −5.1028) (Figures 5A, B, Supplementary Table S3). Further, the prediction model was constructed by combining these 13 factors with NHHR. The AUCs for all-cause and CVD mortality were 0.897 (0.890–0.904) and 0.921 (0.910–0.932), respectively, as seen in Figure 5C, with sensitivity and specificity presented in Supplementary Table S4. Additional DCA and calibration curves also confirmed the clinical applicability of this model (Supplementary Figures S2, S3).

### 3.8 Mediation analysis

**Figure 6:**
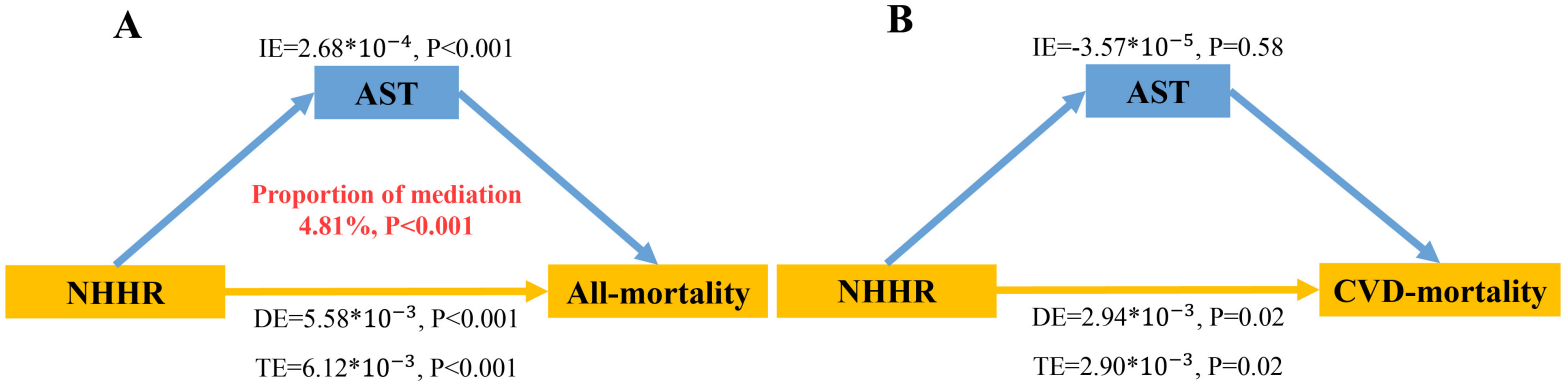
Assessment of the mediating role of AST between NHHR and risk of **(A)** all-cause and **(B)** CVD mortality. NHHR was defined as the independent variable; risk of all-cause or CVD death as the dependent variable; and AST as the mediating variable. IE denotes the mediating effect in which AST plays a role; DE denotes the direct effect of NHHR on the risk of all-cause or CVD mortality; TE denotes the simple total effect of NHHR on the risk of all-cause or CVD mortality; and Proportion of mediation indicates that the proportion of mediation effect in the total effect. IE, indirect effect; DE, direct effect; TE, total effect.

Lastly, considering that abnormal elevation of AST may reflect multiple underlying pathophysiologic states, including hepatocellular injury and myocardial infarction, which may affect the association between NHHR and risk of death. Therefore, we looked for any mediating effects of AST between NHHR and all-cause mortality using mediation analysis. After controlling for variables, NHHR significantly mediated all-cause mortality through AST, as seen in Figure 6 (2.68 * 10^−4^, *P* < 0.001). Moreover, after controlling for AST, NHHR's direct impact on all-cause mortality was still substantial (5.58 * 10^−3^, *P* < 0.001). However, no such effect was observed in CVD mortality risk. This implies that AST acts as a mediator linking NHHR to all-cause mortality, with a mediation degree of approximately 4.81%.

## 4 Discussion

This study utilized NHANES data (1999–2006) to conduct an extensive, multi-ethnic prospective cohort analysis targeting US adults without HHcy. Evaluating the connection between NHHR and the risks of both CVD and all-cause mortality was the goal. Previous clinical practices have suggested that populations with higher Hcy levels are linked to a greater risk of mortality, yet there has been limited focus on individuals with low Hcy levels (32). Therefore, in the NHHcy population, our research is the first to show a link between NHHR and the chances of mortality from CVD and all-cause. With important inflection points at 2.821 and 3.236, respectively, the results show a substantial “U-shaped” link between NHHR and all-cause and CVD mortality. The prediction model's ROC curve further supports the usefulness of NHHR as a gauge of the likelihood of death and clinical prognosis in NHHcy individuals. Additionally, the mediating function of AST in the association of NHHR with mortality outcomes was emphasized by the mediation analysis.

Previous studies have examined the potential link between lipid levels and mortality risk. Ren et al. (33) demonstrated a positive association between triglyceride levels and higher risk of CVD death in patients with heart failure. Similarly, Liang et al. (34), through a large cohort study, concluded that higher risks of mortality was linked to decreased LDL-C levels. Recently, NHHR has gradually gained attention due to its accessibility and wide coverage, being widely used in clinical prediction of various diseases and mortality risks. Mao et al. (20) showed that NHHR is linked to prognosis in myocardial infarction patients and may be utilized as an independent predictor of unfavorable cardiovascular events. These results demonstrate the link between lipid metabolism and physiological health, which subtly validates our findings.

Additionally, accumulating evidence indicates that Hcy is closely associated with nutritional status, lipid metabolism, and mental health. Although the exact biological mechanisms by which Hcy directly affects mortality are still not fully elucidated, the negative correlation between Hcy levels and HDL-C has been well established with the progress of various studies over the past 30 years (35–37). Overall, research on Hcy and lipids and cardiovascular risk can be divided into two phases. In the early controversial phase, Hackam and Anand (38) pointed out that mild to moderate high Hcy levels predispose to atherosclerosis through a review and analysis of 373 relevant studies, and there were pointed out that several meta-analyses proved the relationship between Hcy and CVD, but the support for this relationship is weak and still needs to be supplemented with evidence from large randomized controlled trials. Meanwhile, Tonstad et al. (39) concluded from a large cohort study that in the specific group of familial hypercholesterolemia, total Hcy levels may be associated with the risk of CVD in some patients with familial hypercholesterolemia, and recommended the intake of nutrients related to Hcy metabolism to prevent CVD. However, with the continuous progress of research in recent years, several scholars have confirmed the effect of Hcy on lipid metabolism and CVD. A review by Obeid and Herrmann (40) showed that higher Hcy levels were associated with lower HDL-C levels, which may be related to the inhibition by Hcy of enzymes or molecules involved in the assembly of HDL-C particles. In addition, a recent cross-sectional analysis also indicated that Hcy accelerates lipid accumulation through upregulation of PCSK9, resulting in a significant positive correlation between Hcy levels and risk of severe coronary artery disease (41). We extrapolate that this relationship may be attributable to several pathophysiological mechanisms by which Hcy promotes CVD progression. First, elevated Hcy levels induce endothelial dysfunction, facilitating macrophage accumulation and platelet adhesion—key processes in atherosclerotic plaque development (42, 43). Second, Hcy has been shown to exacerbate systemic inflammation. Given HDL-C's well-established anti-inflammatory properties, lower Hcy levels may preserve HDL-C by reducing inflammatory-mediated consumption (44). Moreover, Hcy is associated with high levels of oxidative stress in the body. And a high oxidative stress state leads to HDL-C dysfunction and enhances atherogenic oxidative modifications and cellular inflammation, which in turn causes vascular endothelial dysfunction (45).

These findings are consistent with ours. More importantly, our findings provide novel evidence that a “U-shaped” association between NHHR and both CVD and all-cause mortality. In particular, below these inflection points, each unit increase in NHHR was linked to a 17.4 and 19% decrease in the probability of mortality from CVD and all-cause, respectively. In contrast, mortality risk increased 6.4 and 11.9% for every unit rise in NHHR over these turning thresholds (*P* for log-likelihood < 0.001). Therefore, for individuals with NHHcy, maintaining NHHR levels between 2.8 and 3.2 would significantly lower the possibility of unfavorable results. Additionally, a number of studies have shown a “U-shaped” association between NHDL-C levels and the risk of both CVD and all-cause mortality in individuals with chronic renal disease and hypertension (46, 47). Another prospective cohort study has also elucidated the “U-shaped” link between unfavorable health consequences and low HDL-C levels (48). Since either too high or low NHDL-C is associated with an increased risk of death, these studies collectively suggest that HDL-C levels must be maintained in the normal range. We speculate that the following reasons might be responsible for the observed differences: (1) The development of atherosclerosis is accelerated by extremely high NHDL-C levels, which results in CVD events (49). (2) Low levels of NHDL-C are often accompanied by low TC levels, an event associated with disease-induced weakness and liver dysfunction, such as hepatitis and liver insufficiency (50). (3) Elevated HDL-C levels are also a significant contributor to low NHDL-C levels, but in most cases, this factor is genetically determined, as seen in primary hyper HDL cholesterolemia, which further increases the risk of CVD mortality (51, 52). Therefore, examining the association between mortality risk and Hcy and NHDL-C levels may improve our understanding of the interplay between lipid metabolism and health outcomes, thereby improving prognosis.

Our examination of subgroups also revealed significant interactions between sex and BMI subgroups with all-cause mortality risk. In particular, people who were older, male, and had a lower BMI were at a higher risk of dying. Increasing age could be related to the previously mentioned association between frailty and extremely low NHDL-C levels, which may increase mortality. It has also been demonstrated in earlier studies that age mediates the relationship between mortality risk, cholesterol levels, and Hcy (53). Additionally, Kaplan-Meier survival curves showed a distinct association linking NHHR levels to mortality risk among various gender groups. Notably, the correlation was found to be opposite between males and females. We hypothesized that unhealthy lifestyles (e.g., smoking, alcohol consumption) in men may exacerbate the risk of dyslipidemia by decreasing HDL-C function to affect the body's sensitivity to lipid metabolism (54). On the other hand, women are more susceptible to estrogen after menopause, and decreased estrogen levels increase cardiovascular risk by decreasing HDL-C synthesis and delaying LDL-C clearance, reducing the ability to compensate for non-HDL-C changes (55). However, additional investigation is still required to validate these hypotheses. Furthermore, we found that AST has a mediation function in the association between NHHR and all-cause mortality risk. According to several research, baseline AST levels are closely related to patient mortality and acute CVD events, particularly excelling in predicting all-cause mortality (56, 57). Researchers speculate that this may be related to underlying disease states, such as liver and kidney diseases, which can cause elevated AST levels and are frequently linked to systemic inflammation, oxidative stress, and apoptosis (58, 59). Nevertheless, there are no conclusive studies showing that AST and NHHR levels are correlated.

This study has several significant strengths: (1) it is the first study to explore the NHHR in a population associated with Hcy; (2) it used a high-quality data source, the NHANES is a standardized collection program under strict governmental regulation, which improves the generalizability of our findings; (3) it considered multiple confounding variables and used multiple filling to deal with the missing data, which enhances the reliability of the data; (4) feature selection and prediction modeling using LASSO regression, and the use of ROC to assess the value of the prediction model, which enhanced the clinical value of our study; and (5) further explored the mediating effect between NHHR and risk of death, which provided preliminary evidence of a potential association between NHHR and mortality.

However, there are some limitations of our study: (1) since this is an observational study, the association between mortality risk and NHHR could not be demonstrated, and a larger prospective cohort study is necessary to further validate it; (2) Hcy data could only be obtained from the database during the investigation period of 1999–2006, and due to the limitations of the sample size, despite adjusting for multiple confounding variables and using a multiple filling method to minimize missing data, some biases may still affect our results; (3) some of the results in the variables of smoking, alcohol consumption, hypertension, and diabetes in the study were derived from self-reported data, and it is difficult to rule out the effect of medical history recall, even though standardized questionnaires have been used by the NHANES to minimize the bias; and (4) since the study sample mainly consisted of the U.S. population, extension of the findings to other racial groups should be further defined. Overall, despite these limitations, this study provides important new information about the relationship between cholesterol levels and health outcomes in the NHHcy population, which informs healthcare professionals' attention to this population and the development of health policies aimed at preventing dyslipidemia and reducing the risk of mortality.

## 5 Conclusion

In summary, NHHR can be used clinically as an effective predictor of mortality risk in the NHHcy population. Its “U-shaped” association with mortality from all-cause and CVD, along with its survival prognostic value across different gender groups, further underscores the importance and necessity of maintaining lipid levels within an optimal range. These findings support the incorporation of NHHR into routine clinical practice and lipid management guidelines as a simple, cost-effective risk stratification tool, particularly in identifying high-risk populations with normal Hcy but poor lipid profiles. Moreover, the predictive model developed in this study requires may help clinicians to improve the assessment of cardiovascular risk by providing personalized monitoring and early intervention in NNHcy populations.

## Data Availability

Publicly available datasets were analyzed in this study. This data can be found here: https://wwwn.cdc.gov/nchs/nhanes/.
