# Supplementary material for: Predictive value of the non-high-density lipoprotein cholesterol to high-density lipoprotein cholesterol ratio (NHHR) for all-cause and cardiovascular mortality with non-hyperhomocysteinemia: evidence from NHANES 1999 to 2006
Source: Front Nutr. 2025 May 20;12:1586558. doi: 10.3389/fnut.2025.1586558 (PMC12129785; doi:10.3389/fnut.2025.1586558)

Supplementary material

Table S1 All covariates and categorical definition of some of them.

Table S2 Multicollinearity assessment of exposure variables with other covariates.

Table S3 Lasso regression analysis results (best log(λ_{1se}) = -5.1028).

Table S4 ROC analysis of NHHR diagnostic performance for all-cause and CVD mortality risk. The model is based on gender, age, race, marital status, PIR, education level, HCY, smoking status, physical activity, stroke, CVD, hypertension and diabetes history were adjusted.

Figure S1 Subgroup interaction effect plots. (A) Interaction between gender in NHHR and all-cause mortality; (B) Interaction between BMI in NHHR and all-cause mortality; (C) Interaction between smoking status in NHHR and CVD mortality; (D) Interaction between alcohol consumption in NHHR and CVD mortality**.**

Figure S2 Performance evaluation of risk prediction models for all-cause mortality. (A) Decision curve analysis (DCA), blue line indicates the net benefit of the prediction model, black line indicates no prediction model was used; (B) Calibration curve, solid line represents the bias-corrected prediction, gray dashed line represents the obvious prediction, and black dashed line represents the ideal prediction.

Figure S3 Performance evaluation of risk prediction models for CVD-cause mortality. (A) Decision curve analysis (DCA), blue line indicates the net benefit of the prediction model, black line indicates no prediction model was used; (B) Calibration curve, solid line represents the bias-corrected prediction, gray dashed line represents the obvious prediction, and black dashed line represents the ideal prediction.

Table S1 All covariates and categorical definition of some of them.

Covariates		Definition
**Demographic variables**	age	\
	gender	Male; Female
	race	Mexican American, Other Hispanic, On-Hispanic White, On-Hispanic Black, Other Race
	marital status	Married or living with a partner; Widowed/divorced/separated; Never married
	education level	Less than high school; High school or equivalent; College graduate or above
	poverty income ratio (PIR)	≤1.3, 1.3-3.5, ≥3.5
	follow-up period (months)	\
	mortality	All-cause mortality
**Examination variables**	body mass index (BMI)	Normal, <25 kg/m^2; Overweight, 25-30 kg/m^2; Obese, ≥30 kg/m^2
	systolic blood pressure (SBP; mmHg)	\
	diastolic blood pressure (DBP; mmHg)	\
**Laboratory variables**	Homocysteine (Hcy; umol/L)	\
	aspartate aminotransferase (AST; U/L)	\
	total cholesterol (TC; mmol/L)	\
	high-density lipoprotein cholesterol (HDL-C, mmol/L)	\
	Folate serum (nmol/L)	\
	Folate erythrocyte (nmol/L)	\
	Vitamin B12 (IQR, pmol/L)	\
**Lifestyle variables**	smoking status	Yes: having smoked a minimum of 100 cigarettes throughout their life
	alcohol consumption	Yes: minimum of 12 alcoholic drinks annually
	diabetes	Yes: Self-reported diabetes or fasting blood glucose >7.0 mmol/L or glycosylated hemoglobin >6.5%; Prediabetes: Self-reported prediabetes or fasting blood glucose between 6.1 and 7.0 mmol/L
	hypertension	Yes: Self-reported hypertension or mean systolic blood pressure ≥140 mmHg or mean diastolic blood pressure ≥90 mmHg
	cardiovascular disease (CVD)	Yes: Self-reported coronary heart disease, myocardial infarction, congestive heart failure and angina pectoris
	physical activity	Yes: work involves moderate-intensity activities that result in small increases in respiration or heart rate
	stroke	Yes: Self-reported stroke

Table S2 Multicollinearity assessment of exposure variables with other covariates.

Variable	VIF value
Age	1.885273
Gender	1.331667
Race/ethnicity	1.155204
Education level	1.427931
Marital status	1.131324
PIR	1.366052
BMI	1.151952
AST	1.013852
Folate serum	1.296729
Folate erythrocyte	1.356755
Vitamin B12	1.008239
HCY	1.413907
Smoking status	1.137502
Alcohol consumption	1.217086
Diabetes	1.080474
Hypertension	1.373052
Cardiovascular disease	1.132657
Physical activity	1.110695
Stroke	1.053445
NHHR	1.172511

Abbreviation: PIR, poverty income ratio; BMI, body mass index; CVD, cardiovascular disease; AST, aspartate aminotransferase; HCY, homocysteine.

Table S3 Lasso regression analysis results (best log(λ_{1se}) = -5.1028).

**Characteristic**	**r**	**Characteristic**	**r**
Gender	-5.3277	Physical activity	0.2275
Age	0.0912	Stroke	-0.6036
Race	-0.1873	CVD	0.4791
Marital status	-0.2215	AST	-
PIR	-0.1305	Hypertension	-0.1318
Education level	-0.1479	Diabetes	0.2932
BMI	-	HCY	0.0330
Alcohol consumption	-	Folate serum	-
Smoking status	4.0659	Folate erythrocyte	-
Vitamin B12	-		

Abbreviation: PIR, poverty income ratio; BMI, body mass index; CVD, cardiovascular disease; AST, aspartate aminotransferase; HCY, homocysteine.

Table S4 ROC analysis of NHHR diagnostic performance for all-cause and CVD mortality risk. The model is based on gender, age, race, marital status, PIR, education level, HCY, smoking status, physical activity, stroke, CVD, hypertension and diabetes history were adjusted.

	AUC (95%CI)	Accuracy (95%CI)	Sensitivity (95%CI)	Specificity (95%CI)	PPV (95%CI)	NPV (95%CI)	Cut off	Youden index
All-cause mortality	0.897 (0.890-0.904)	0.810 (0.804-0.817)	0.802 (0.794 - 0.809)	0.842 (0.829 - 0.855)	0.951 (0.946 - 0.955)	0.528 (0.514 - 0.542)	0.193	0.644
CVD-cause mortality	0.921 (0.910-0.932)	0.857 (0.850-0.864)	0.857 (0.850 - 0.864)	0.852 (0.825 - 0.879)	0.989 (0.986 - 0.991)	0.286 (0.266 - 0.306)	0.074	0.709

Figure S1 Subgroup interaction effect plots. (A) Interaction between gender in NHHR and all-cause mortality; (B) Interaction between BMI in NHHR and all-cause mortality; (C) Interaction between smoking status in NHHR and CVD mortality; (D) Interaction between alcohol consumption in NHHR and CVD mortality.

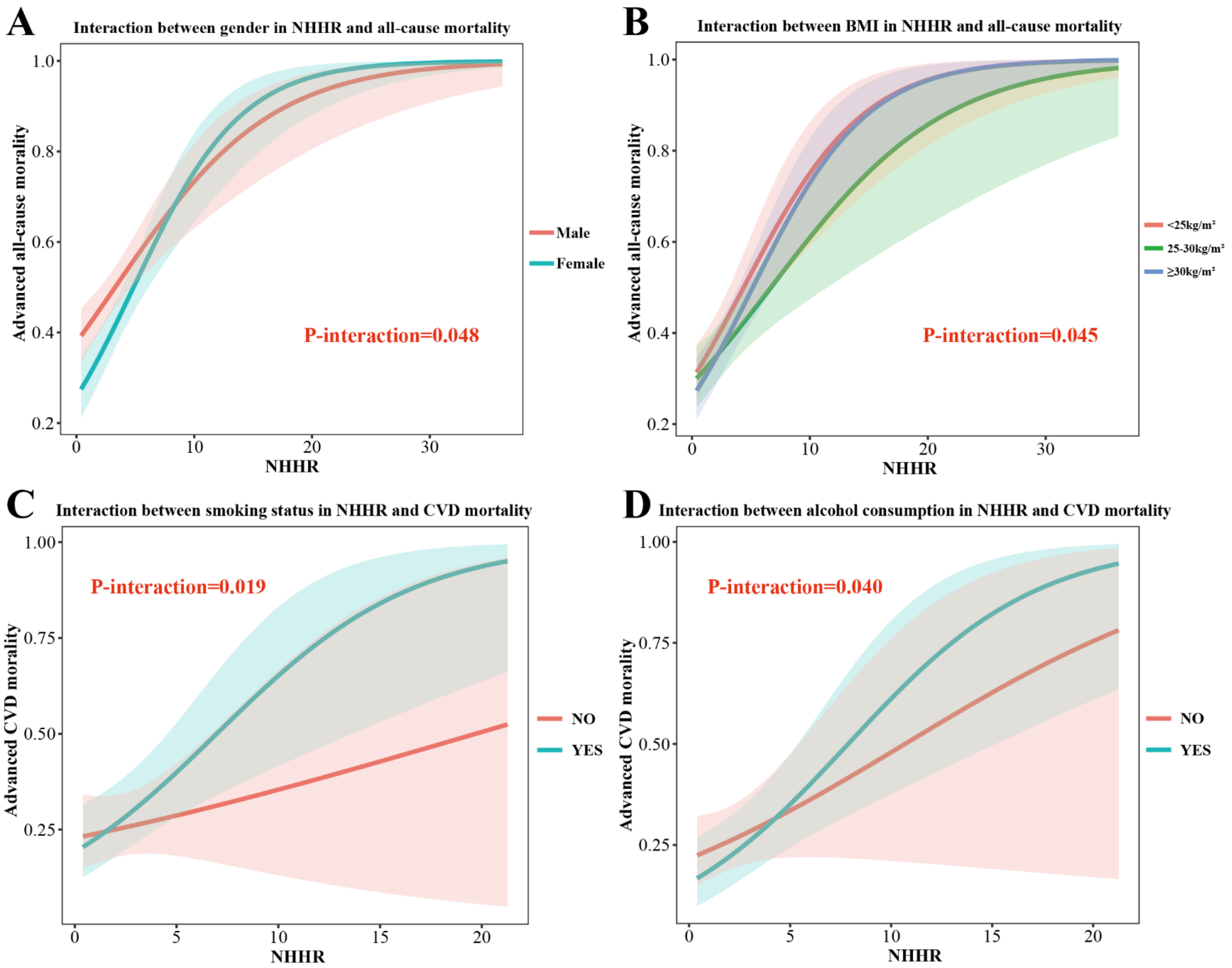

Figure S2 Performance evaluation of risk prediction models for all-cause mortality. (A) Decision curve analysis (DCA), blue line indicates the net benefit of the prediction model, black line indicates no prediction model was used; (B) Calibration curve, solid line represents the bias-corrected prediction, gray dashed line represents the obvious prediction, and black dashed line represents the ideal prediction.

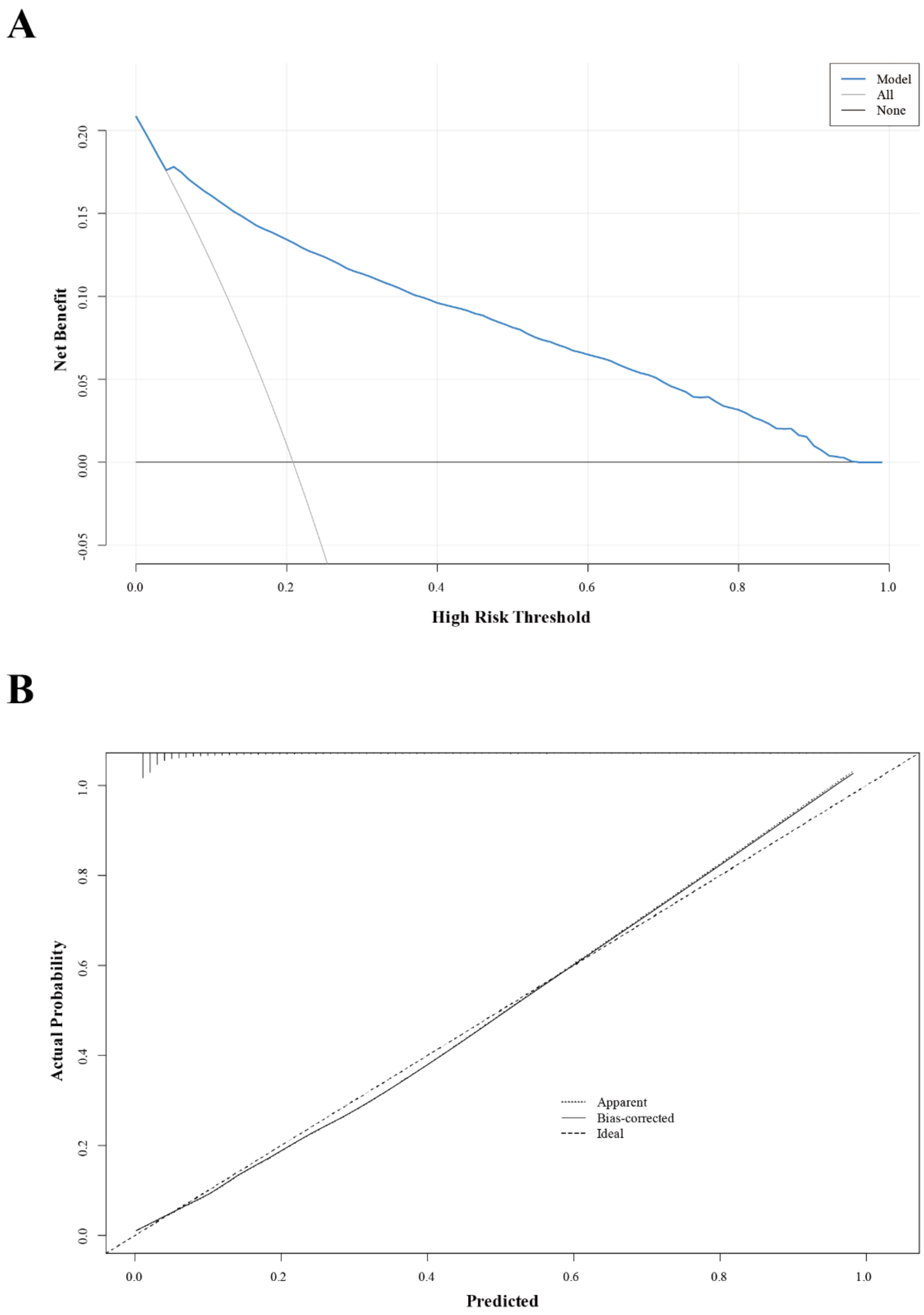

Figure S3 Performance evaluation of risk prediction models for CVD-cause mortality. (A) Decision curve analysis (DCA), blue line indicates the net benefit of the prediction model, black line indicates no prediction model was used; (B) Calibration curve, solid line represents the bias-corrected prediction, gray dashed line represents the obvious prediction, and black dashed line represents the ideal prediction.

A

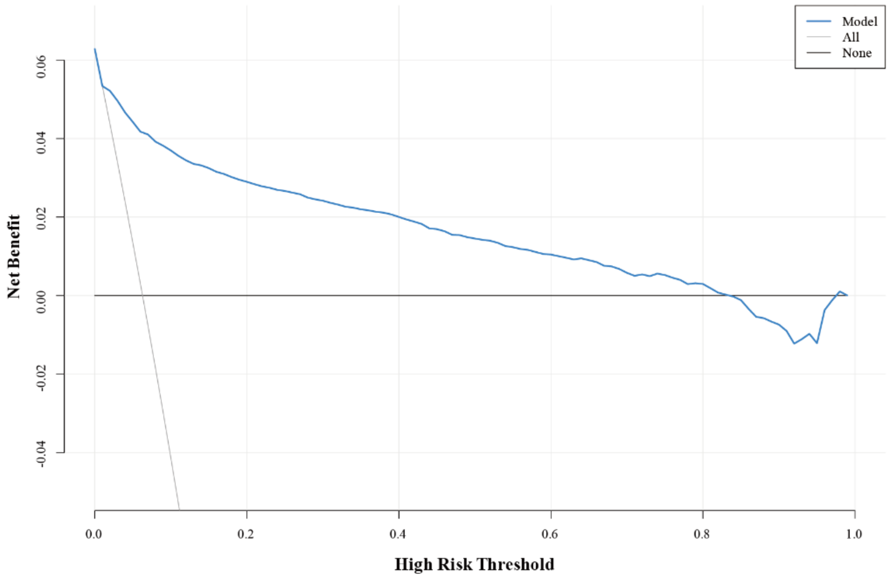

B

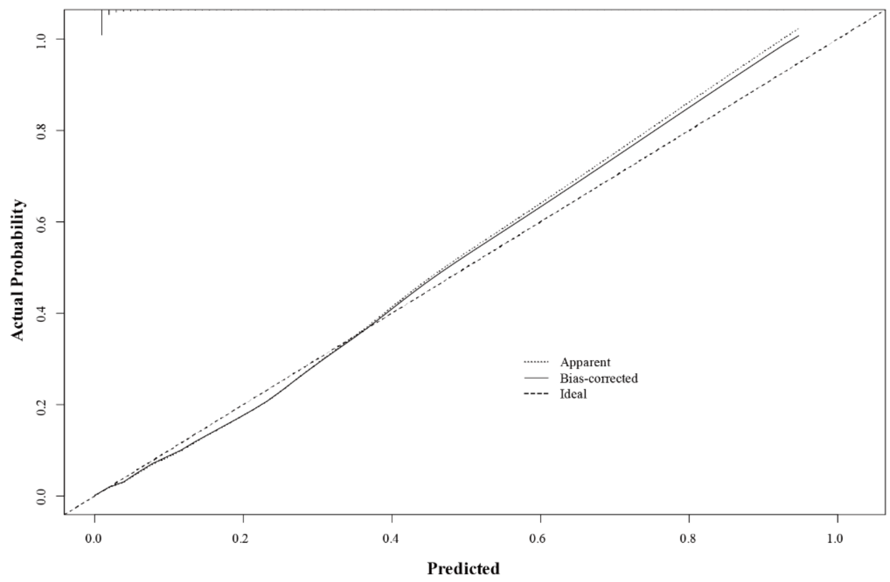

Supplement: Supplementary file 1 [file Data_Sheet_1.pdf]
